# Endoscopic diagnosis of immunoglobulin G4‐related sclerosing cholangitis

**DOI:** 10.1111/den.15039

**Published:** 2025-04-21

**Authors:** Itaru Naitoh, Michihiro Yoshida, Takahiro Nakazawa

**Affiliations:** ^1^ Department of Gastroenterology Nagoya City University Midori Municipal Hospital Aichi Japan; ^2^ Department of Gastroenterology Nagoya City University Graduate School of Medical Sciences Aichi Japan

**Keywords:** autoimmune pancreatitis, endoscopic retrograde cholangiopancreatography, endoscopic ultrasonography, IgG4‐related sclerosing cholangitis, intraductal ultrasonography

## Abstract

Immunoglobulin G4 (IgG4)‐related sclerosing cholangitis (IgG4‐SC) is a distinct form of sclerosing cholangitis frequently associated with autoimmune pancreatitis and is recognized as a biliary manifestation of IgG4‐related disease. Endoscopic retrograde cholangiopancreatography (ERCP) and endoscopic ultrasonography (EUS) are key diagnostic modalities for IgG4‐SC. Cholangiocarcinoma and primary sclerosing cholangitis (PSC) are significant mimickers of IgG4‐SC. ERCP is employed to evaluate narrowing of the bile duct, with cholangiograms of IgG4‐SC classified into four types. This cholangiographic classification is crucial for differential diagnosis. Characteristic cholangiographic findings of IgG4‐SC include diffuse or segmental strictures of the intrahepatic or extrahepatic bile ducts and intrahepatic strictures associated with autoimmune pancreatitis. ERCP is particularly useful for differentiating IgG4‐SC from PSC because their cholangiographic features differ. EUS and intraductal ultrasonography (IDUS) are used to assess thickening of the bile duct wall. Characteristic IDUS findings in IgG4‐SC include circular and symmetrical wall thickening, smooth outer and inner margins, and homogeneous internal echoes at stricture sites. Additionally, bile duct wall thickening at nonstricture sites is a typical IDUS feature of IgG4‐SC. Bile duct biopsy is used to evaluate pathological findings, although its diagnostic yield for IgG4‐SC is limited; its primary role is to exclude malignant biliary strictures. Duodenal papilla biopsy serves as a supplementary diagnostic tool for IgG4‐SC. EUS and tissue acquisition also aid in diagnosing autoimmune pancreatitis as part of other organ involvement. Thus, endoscopic techniques play critical roles in the diagnosis of IgG4‐SC.

## INTRODUCTION

Immunoglobulin G4 (IgG4)‐related sclerosing cholangitis (IgG4‐SC) is a distinct form of sclerosing cholangitis with an unclear pathogenic mechanism. It is characterized by elevated serum IgG4 levels and marked lymphoplasmacytic infiltration and fibrosis, with the presence of IgG4‐positive plasma cells and lymphocytes. Frequently associated with type 1 autoimmune pancreatitis (AIP), IgG4‐SC is recognized as a biliary manifestation of IgG4‐related disease (IgG4‐RD). Clinically, IgG4‐SC presents with biliary strictures, thickening of the bile duct wall, and a favorable response to steroid therapy.^1^, ^2^, ^3^

Immunoglobulin G4‐related sclerosing cholangitis presents with various types of cholangiograms, many of which share similarities with the cholangiographic features of primary sclerosing cholangitis (PSC), cholangiocarcinoma (CCA), and pancreatic cancer. However, the treatment and prognosis of IgG4‐SC differ significantly from those of these progressive or malignant diseases. Therefore, accurately distinguishing IgG4‐SC from these mimickers is crucial. Cholangiographic classification based on the location of the biliary stricture is a valuable tool for the differential diagnosis of IgG4‐SC. In clinical practice, the diagnosis of IgG4‐SC is guided by the Clinical Diagnostic Criteria 2020 (IgG4‐SC2020).^3^

Endoscopic modalities play a critical role in the diagnosis of IgG4‐SC and its differentiation from other mimickers. Endoscopic retrograde cholangiopancreatography (ERCP) and endoscopic ultrasonography (EUS) are two major endoscopic modalities in the diagnosis of IgG4‐SC. ERCP‐related procedures include biliary intraductal ultrasonography (IDUS), transpapillary bile duct biopsy and duodenal papilla biopsy, and peroral cholangioscopy (POCS). EUS‐related procedures include EUS‐guided tissue acquisition (EUS‐TA). Several review articles of IgG4‐SC have been published to date.^4^, ^5^, ^6^ However, there is a scarcity of recently published studies presenting novel findings and evidence regarding the endoscopic diagnosis of IgG4‐SC. Therefore, we focused on the current roles of endoscopic modalities in the diagnosis of IgG4‐SC in this literature review.

## DIAGNOSTIC CRITERIA FOR IgG4‐SC

The clinical diagnostic criteria 2012 (IgG4‐SC2012) were first proposed in Japan.^1^ The IgG4‐SC2012 consist of four criteria: characteristic biliary imaging findings, elevation of the serum IgG4 concentration, coexistence with other IgG4‐RDs, and characteristic histopathological features. The effectiveness of steroid therapy is an optional additional criterion for confirming an accurate diagnosis of IgG4‐SC. The IgG4‐SC2012 have been widely used in clinical practice since their introduction.

The clinical diagnostic criteria 2020 (IgG4‐SC2020) were proposed as a revision of the IgG4‐SC2012 in 2020.^3^ The IgG4‐SC2020 comprise six criteria: narrowing of the intrahepatic and/or extrahepatic bile duct, thickening of the bile duct wall, serological findings, pathological findings, coexistence with other IgG4‐RDs, and the effectiveness of steroid therapy.

In the IgG4‐SC2020, endoscopic modalities can be used to evaluate diagnostic items. Endoscopic retrograde cholangiography (ERC) is used to assess narrowing of the bile duct. EUS and IDUS are employed to evaluate thickening of the bile duct wall. EUS‐TA for the bile duct and transpapillary bile duct biopsy are used to assess the pathological findings of IgG4‐SC. Endoscopic retrograde pancreatography, EUS, and EUS‐TA for the pancreas are utilized for diagnosing AIP as other organ involvement (OOI) in IgG4‐SC. Although not included in the IgG4‐SC2020, duodenal papilla biopsy serves as a supplemental tool for diagnosing IgG4‐SC.

## CHOLANGIOGRAPHIC CLASSIFICATION OF IgG4‐SC

**Figure 1:**
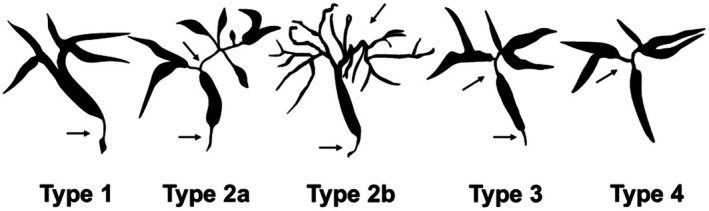
Cholangiographic classification of immunoglobulin G4‐related sclerosing cholangitis. Adapted from Ohara *et al*. 2012,^1^ with permission from John Wiley and Sons. Type 1: Stenosis is confined to the lower common bile duct. Type 2: Stenosis is diffusely distributed throughout the intrahepatic and extrahepatic bile ducts. Type 2a: Stricture of the intrahepatic bile ducts with prestenotic dilation. Type 2b: Stricture of the intrahepatic bile ducts without prestenotic dilation and with reduced bile duct branches. Type 3: Stenosis involves both the hilar hepatic and lower common bile ducts. Type 4: Stenosis is limited to the hilar hepatic bile ducts. Arrows indicate stenosis of the bile ducts.

Cholangiographic classification is crucial for the differential diagnosis of IgG4‐SC. This classification is included in the IgG4‐SC2020 criteria^3^ and is described in the algorithm for diagnosing IgG4‐SC in the clinical practice guidelines for IgG4‐SC.^2^ Cholangiograms of IgG4‐SC are categorized into four types based on the stricture sites for differential diagnosis^7^ (Fig. 1). In a Japanese nationwide survey,^8^ the incidence levels of type 1, type 2a, type 2b, type 3, and type 4 cholangiograms were reported as 62.9%, 7.6%, 5.7%, 9.6%, and 10.3%, respectively. Type 1 was the most common cholangiogram type in IgG4‐SC (62.9%) and in IgG4‐SC associated with AIP (69.9%), whereas type 4 was the most prevalent type in IgG4‐SC not associated with AIP (30.9%).

In type 1 IgG4‐SC, biliary stricture is present only in the distal common bile duct, and it should be differentiated from pancreatic cancer, distal CCA, and chronic pancreatitis. In type 2 IgG4‐SC, diffuse strictures are observed throughout the intrahepatic and extrahepatic bile ducts, and this type should be distinguished from PSC. Type 2 IgG4‐SC is further subdivided into two subtypes: type 2a, characterized by intrahepatic bile duct strictures with prestenotic dilation, and type 2b, characterized by intrahepatic bile duct strictures without prestenotic dilation and with reduced bile duct branches. Type 3 IgG4‐SC involves strictures in both the hilar hepatic and distal common bile ducts, whereas type 4 IgG4‐SC presents with strictures localized to the hilar hepatic bile ducts. Types 3 and 4 IgG4‐SC need to be differentiated from hilar CCA. Rare cases of IgG4‐SC that do not fit into the four cholangiographic classification types involve strictures in the extrahepatic bile ducts, excluding the intrapancreatic bile duct. According to the IgG4‐SC2020, such cases should be categorized as type 4 IgG4‐SC.^3^

## ERCP

Diagnostic ERCP has been used for diagnosing AIP and IgG4‐SC in Japan but is rarely used in Western countries. According to the IgG4‐SC2012,^1^ direct cholangiography, such as ERC or percutaneous transhepatic cholangiography, is mandatory for evaluating cholangiograms. However, with recent advancements in imaging quality and the reduced invasiveness of magnetic resonance cholangiography (MRC), MRC has become the standard procedure. As a result, MRC is included as a modality alongside direct cholangiography for assessing narrowing of the bile duct in the IgG4‐SC2020.^3^ The visualization of biliary stricture sites is of a higher quality with ERC than with MRC. However, ERC is associated with potential adverse events, including post‐ERCP pancreatitis (PEP), bleeding, perforation, and cholangitis. Among these, PEP is the most common and can occasionally lead to fatal outcomes. According to a recent systematic review of randomized controlled trials, the overall cumulative incidence of PEP was 10.2%, with an incidence of severe PEP and mortality at 0.5% and 0.2%, respectively.^9^ Our previous study demonstrated practical evidence showing that the incidence of PEP was lower in patients with AIP than in normal controls (1.2% vs. 5.4%, respectively).^10^ Despite this relatively low incidence of PEP in IgG4‐SC, it is crucial to avoid unnecessary ERC in its diagnosis because of the potential risk of severe adverse events. ERC is necessary in cases where pathologic approaches should be considered for the differential diagnosis or biliary drainage is unnecessary. On the contrary, ERC can be avoided in cases where a definite diagnosis of IgG4‐SC is obtained on the basis of IgG4‐SC2020, and pathologic approach to the biliary stricture in unnecessary, and mild jaundice without cholangitis is observed. For example, cases with distal biliary stricture associated with elevated serum IgG4 level and diffuse enlargement of the pancreas, or definitive diagnosis of AIP has been obtained by EUS‐TA.

**Figure 2:**
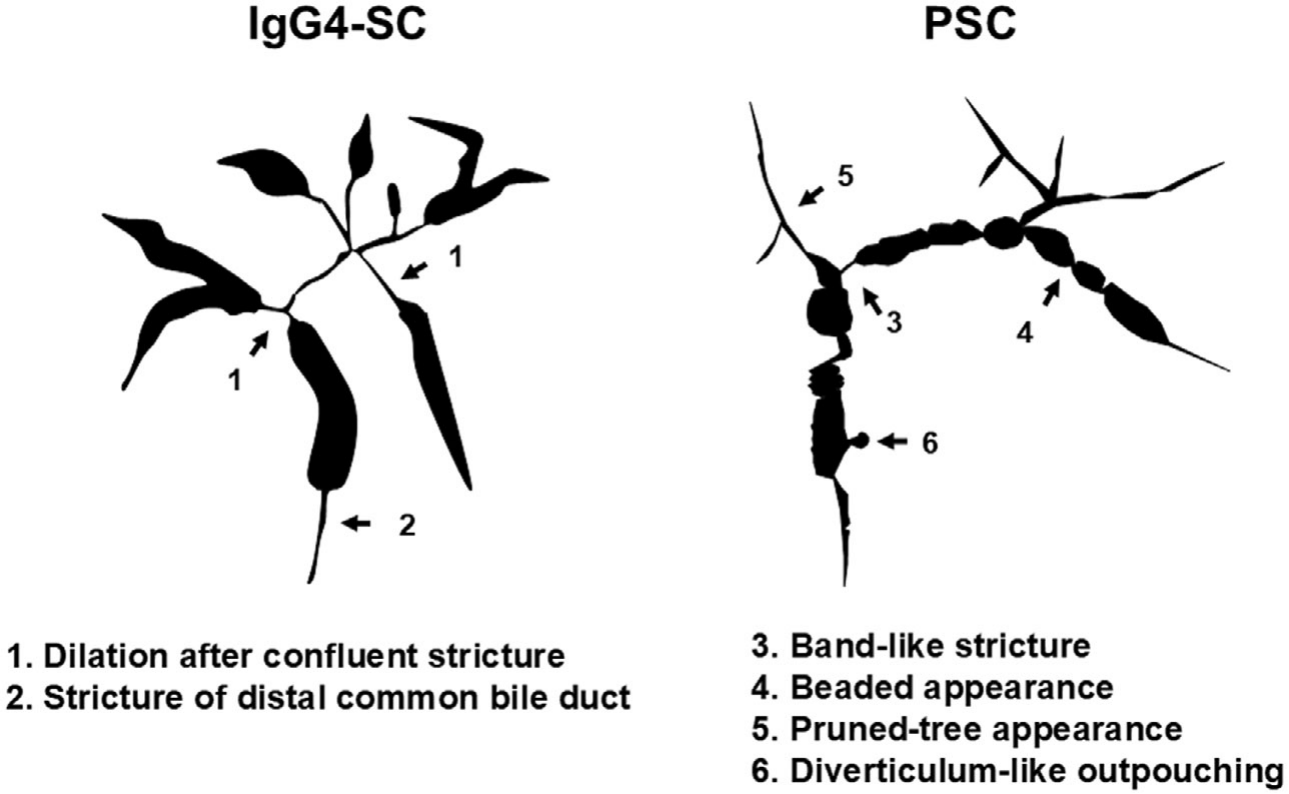
The characteristic features of cholangiograms between immunoglobulin G4‐related sclerosing cholangitis (IgG4‐SC) and primary sclerosing cholangitis (PSC) on endoscopic retrograde cholangiography. Adapted from Ohara *et al*. 2012,^1^ with permission from John Wiley and Sons.

Primary sclerosing cholangitis is an important mimicker of type 2 IgG4‐SC because of the similarity of cholangiograms between these conditions. We previously reported that ERC is useful for differentiating IgG4‐SC from PSC because the detailed features of the cholangiograms differ between the two on ERC.^11^ The cholangiogram of IgG4‐SC is characterized by relatively long strictures, often accompanied by simple dilation following a confluent stricture. Intrapancreatic biliary strictures are commonly observed because of the frequent association with AIP. By contrast, band‐like strictures (1–2 mm), a beaded or pruned‐tree appearance, and diverticulum‐like outpouchings are characteristic of PSC cholangiograms (Fig. 2). ERC is superior to MRC in visualizing these detailed differences because MRC does not provide the same level of resolution. However, as mentioned earlier, MRC has become the standard procedure for evaluating cholangiograms because of improvements in image quality. The diagnostic accuracy of MRC is comparable to that of ERC in diagnosing PSC,^12^ and the diagnostic criteria for PSC encourage the use of MRC as the initial diagnostic modality. Therefore, unnecessary ERC should be avoided when high‐quality cholangiograms can be obtained via MRC. We commonly perform ERC to conduct additional ERC‐related procedures, as described below.

## IDUS

**Figure 3:**
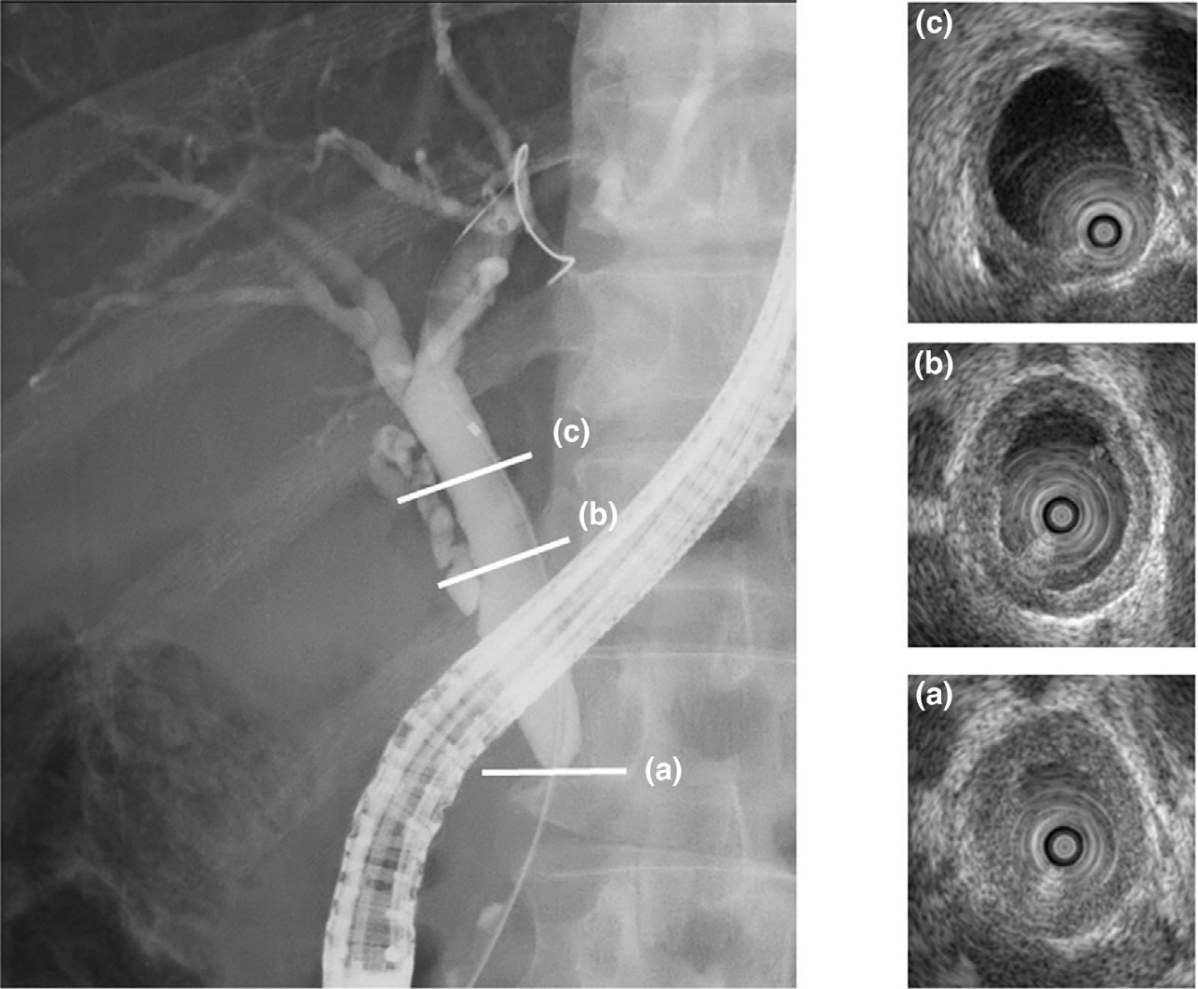
Intraductal ultrasonography (IDUS) findings of immunoglobulin G4 (IgG4)‐related sclerosing cholangitis (IgG4‐SC). Endoscopic retrograde cholangiography showing intrapancreatic strictures. (a) IDUS reveals wall thickening in the intrapancreatic stricture. IDUS findings include circular‐symmetrical wall thickening with smooth inner and outer margins. (b) IDUS demonstrates wall thickening in the middle bile duct at a nonstricture site. (c) IDUS shows wall thickening in the hilar bile duct at a nonstricture site.

**Figure 4:**
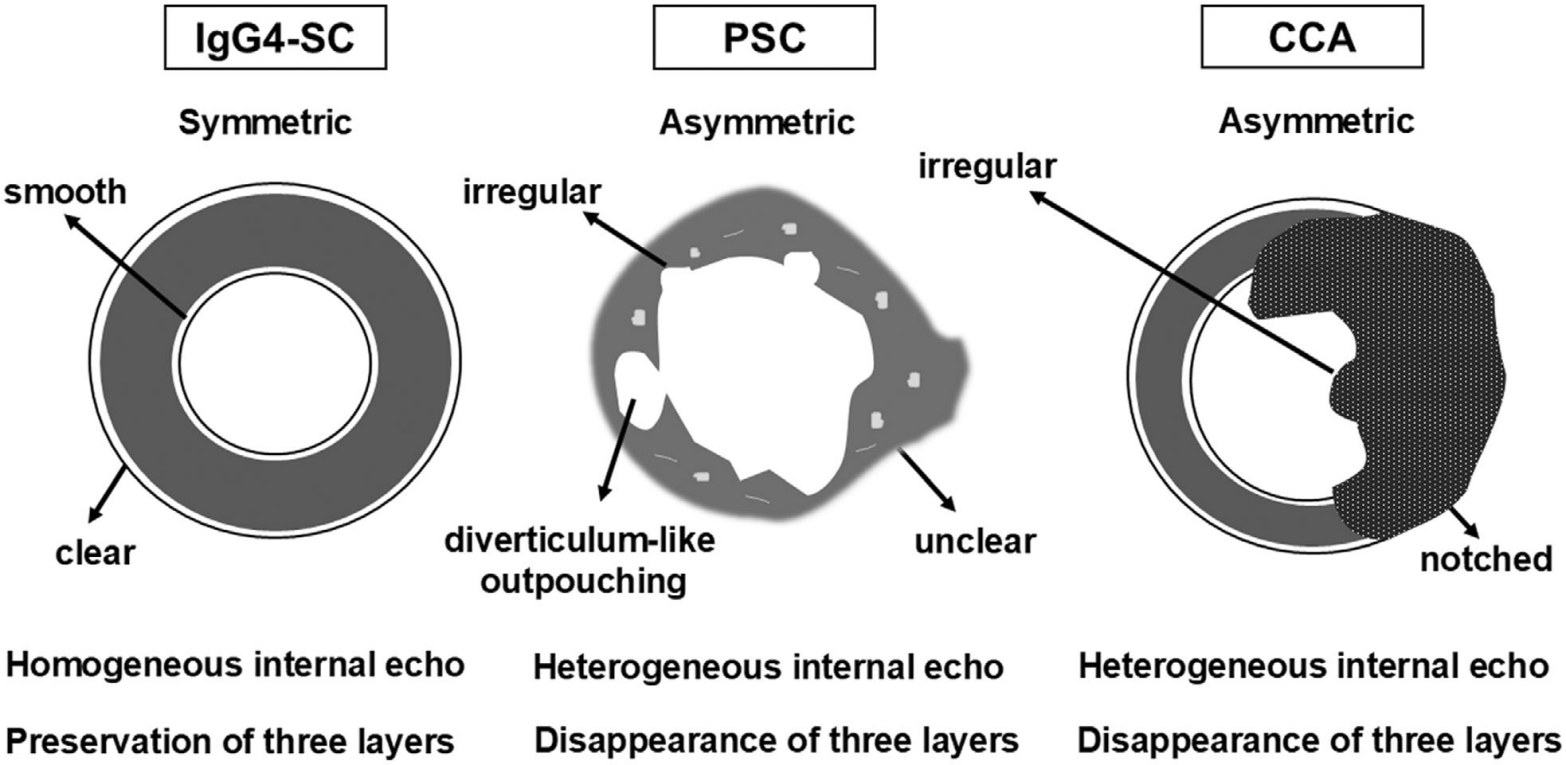
Intraductal ultrasonographic findings in the biliary stricture among immunoglobulin G4‐related sclerosing cholangitis (IgG4‐SC), primary sclerosing cholangitis (PSC), and cholangiocarcinoma (CCA).

Intraductal ultrasonography is a reliable procedure for evaluating the bile duct during ERC because it provides high‐resolution images of the bile duct wall. IDUS should be performed before initial biliary drainage because mechanical inflammation of the bile duct wall can occur following drainage. Thickening of the bile duct wall is included as a diagnostic criterion in the IgG4‐SC2020.^3^ Characteristic IDUS findings for IgG4‐SC include circular‐symmetrical wall thickening, smooth outer and inner margins, and a homogeneous internal echo at the biliary stricture site^13^, ^14^, ^15^, ^16^, ^17^ (Fig. 3). By contrast, PSC is characterized by IDUS findings of circular‐asymmetrical wall thickening, irregular inner margins, unclear outer margins, diverticulum‐like outpouchings, a heterogeneous internal echo, and the disappearance of the three‐layered structure.^18^ IDUS evaluation of wall thickening at the stricture site is particularly useful for differentiating IgG4‐SC from PSC. Thickening of the bile duct wall at nonstricture sites is another characteristic IDUS finding of IgG4‐SC. In IgG4‐SC, the thickening spreads continuously from the intrapancreatic bile duct to the hilar bile duct.^13^, ^15^, ^18^ However, such thickening at nonstricture sites is not typically observed in CCA. IDUS findings at nonstricture sites are therefore useful for distinguishing IgG4‐SC from CCA.^13^ The IDUS findings for IgG4‐SC, PSC, and CCA are summarized in Figure 4. IDUS is a valuable tool for differentiating IgG4‐SC from PSC and CCA. However, as previously mentioned, caution is needed because of the risks associated with ERCP.

## BILE DUCT BIOPSY

Bile duct biopsy is essential for obtaining histological evidence of malignant biliary strictures and is performed to differentiate indeterminate biliary strictures. IgG4 immunostaining is valuable for the histological diagnosis of IgG4‐SC, with reported sensitivities ranging from 0% to 88%.^13^, ^19^, ^20^, ^21^ Pathological findings incorporated into IgG4‐SC2020 include marked lymphoplasmacytic infiltration and fibrosis, more than 10 IgG4‐positive plasma cells per high‐power field (HPF), storiform fibrosis, and obliterative phlebitis.^3^ However, a Japanese nationwide survey reported the rates of these findings in bile duct biopsy samples as 32.9%, 16.9%, 0.6%, and 0.0%, respectively.^8^ This low sensitivity is attributed to the histopathological characteristics of IgG4‐SC, where lymphoplasmacytic infiltration and fibrosis are located in the bile duct wall stroma beneath normal epithelium. Consequently, obtaining a sufficiently large sample containing the bile duct stroma for histological diagnosis using small biopsy forceps is challenging.

Cholangiocarcinoma is a significant mimicker of IgG4‐SC, making bile duct biopsy crucial for diagnosing CCA. According to a systematic review and meta‐analysis, the pooled sensitivity of bile duct biopsy in detecting malignant biliary strictures was 48.1%.^22^ In our retrospective study, the sensitivity of bile duct biopsy specifically for diagnosing CCA was 82.0%.^23^ Although bile duct biopsy may not be effective for obtaining a histopathological diagnosis of IgG4‐SC, it plays a vital role in excluding malignant biliary strictures, such as those caused by CCA.

Bile duct cytology is another method used to diagnose malignant biliary strictures. In a Japanese nationwide survey,^8^ brush cytology samples from 369 patients with IgG4‐SC were categorized as class 4 in 0.8% and class 5 in 0.5%, with 5 (1.3%) patients being misdiagnosed with malignancy based on brush cytology. Therefore, caution is required when malignancy is suspected from brush cytology samples in the diagnosis of IgG4‐SC.

## POCS

**Figure 5:**
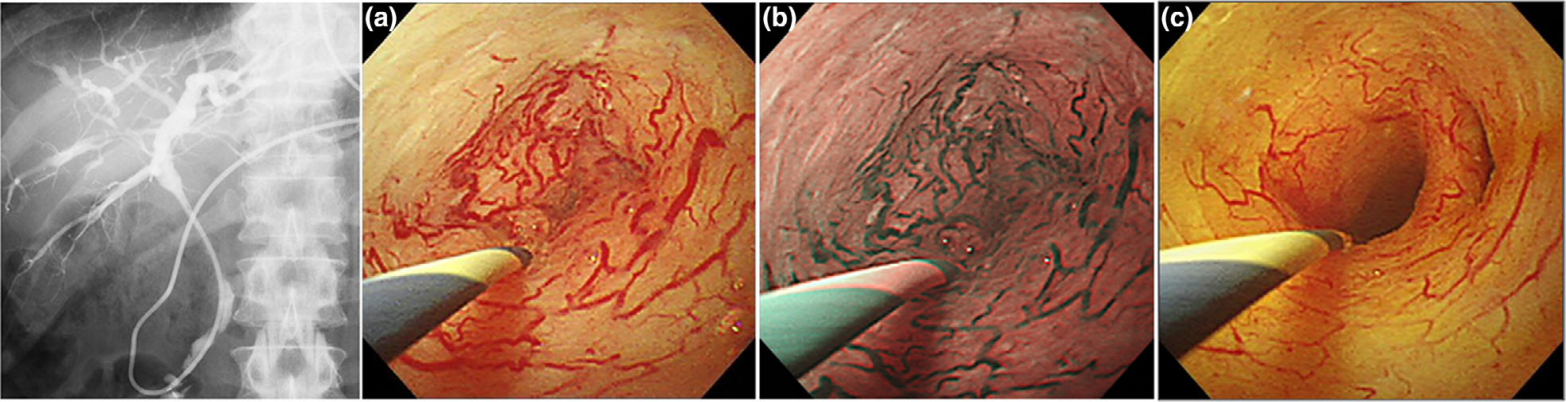
Peroral cholangioscopy (POCS) findings of immunoglobulin G4 (IgG4)‐related sclerosing cholangitis (IgG4‐SC). Images courtesy of Dr. Yasutaka Ishii, Hiroshima University. Endoscopic retrograde cholangiography showing intrahepatic and hilar strictures before steroid therapy. POCS reveals a smooth mucosal surface, dilated vessels without caliber alteration, and an absence of easy bleeding in (a) white light imaging and (b) in narrow‐band imaging before steroid therapy. (c) Biliary stricture and dilated vessels are improved after steroid therapy in white light imaging.

Peroral cholangioscopy is performed to differentiate between benign and malignant biliary strictures. Ishii *et al*.^24^ reported that characteristic POCS findings for IgG4‐SC at stricture sites include a smooth mucosal surface, dilated vessels without caliber alteration or disruption, and an absence of easy bleeding, making these findings highly useful for distinguishing IgG4‐SC from CCA (Fig. 5). Dilated and tortuous vessels have also been identified as characteristic POCS findings for IgG4‐SC.^25^, ^26^ POCS is a valuable modality for differentiating IgG4‐SC from CCA. Among these, isolated IgG4‐SC, which occurs in the absence of AIP, presents the greatest diagnostic challenge in distinguishing it from CCA. Therefore, POCS is particularly indicated in cases of isolated IgG4‐SC or when the association with AIP has not been clearly established. After malignancy has been excluded through bile duct biopsy or cytology, assessing changes in mucosal morphology and vascular patterns with POCS before and after steroid treatment provides additional valuable diagnostic information.

Image‐enhanced endoscopy (IEE) has significantly advanced the diagnosis and treatment of gastrointestinal diseases and has recently been applied to pancreatobiliary endoscopy. Narrow‐band imaging (NBI), texture and color enhancement imaging, and red dichromatic imaging are among the IEE modalities utilized in POCS. There are various types of POCS systems, and the capability to use IEE is available with Olympus's mother–baby‐type POCS. NBI enhances mucosal structures and vascular patterns, and its utility in POCS has been documented, particularly in the diagnosis of intraductal neoplasms of the bile duct.^27^ NBI might be promising for detecting subtle mucosal and vascular changes in the diagnosis of IgG4‐SC, as dilated and tortuous vessels without caliber alteration or disruption are characteristic POCS findings. Further investigations employing emerging IEE techniques, such as texture and color enhancement imaging and red dichromatic imaging, are warranted to elucidate the POCS findings of IgG4‐SC.

## DUODENAL PAPILLA BIOPSY

Swollen duodenal papilla with abundant IgG4‐positive plasma cells is observed particularly in cases of AIP with pancreatic head involvement. The international consensus diagnostic criteria include duodenal papilla biopsy as an optional diagnostic method for AIP.^28^ In our previous study, pancreatic head involvement, intrapancreatic IgG4‐SC, and swollen duodenal papilla were significantly associated with positive IgG4 immunostaining in duodenal papilla samples.^29^ Histopathological analysis of duodenal papilla biopsy specimens using IgG4 immunostaining is a valuable supplemental tool for diagnosing IgG4‐SC.^15^, ^29^, ^30^, ^31^, ^32^ Duodenal papilla biopsy with IgG4 immunostaining is useful for the differential diagnosis of IgG4‐SC from other mimickers. In a Japanese nationwide survey,^8^ swollen duodenal papillae were found in 25.4% of IgG4‐SC cases, and more than 10 IgG4‐positive plasma cells per HPF were observed in 36.8% of cases. The incidence of more than 10 IgG4‐positive plasma cells per HPF was higher in duodenal papilla biopsies than in bile duct biopsies (36.8% vs. 16.9%) and was also observed in 23.8% of IgG4‐SC cases without AIP. This indicates that papilla biopsy is useful not only for diagnosing IgG4‐SC with AIP but also for isolated IgG4‐SC. A systematic review and meta‐analysis reported a pooled sensitivity of 51% and specificity of 97% for IgG4 immunostaining in biliary and duodenal papilla biopsy specimens for the diagnosis of AIP.^33^ Duodenal papilla biopsy is a simple and technically easy procedure compared to bile duct biopsy. Its current role is as a supplemental tool in diagnosing IgG4‐SC.

## EUS

The EUS finding of bile duct wall thickening suggests the presence of IgG4‐SC. No study has reported the detailed EUS findings of IgG4‐SC to date. IUDS findings of IgG4‐SC might be helpful in the consideration of EUS findings because characteristic findings of IgG4‐SC have been reported, as mentioned previously. In a Japanese nationwide survey,^8^ wall thickening at nonstricture sites was observed in 73.8% of IgG4‐SC. Further study of EUS findings of IgG4‐SC is needed because EUS is a less invasive procedure for the detailed evaluation of thickening of the bile duct wall, which is incorporated in the diagnostic items of IgG4‐SC2020.^3^

The existence of AIP is crucial in the diagnosis of IgG4‐SC because most cases of IgG4‐SC are associated with AIP, which is included as a key OOI in the diagnostic criteria of the IgG4‐SC2020.^3^ In the previously mentioned Japanese nationwide survey,^8^ AIP was associated with 83.7% of IgG4‐SC cases. EUS is a valuable modality for diagnosing AIP. Although diffuse‐type AIP is generally straightforward to diagnose, focal‐type AIP can be challenging to differentiate from pancreatic cancer. Several studies have demonstrated the utility of conventional EUS, contrast‐enhanced harmonic EUS, and EUS elastography in distinguishing AIP from pancreatic cancer.^34^, ^35^, ^36^, ^37^ Therefore, the current role of EUS in IgG4‐SC diagnosis is that of identifying AIP as an OOI.

## EUS‐TA

Endoscopic ultrasonography‐guided tissue acquisition is not commonly performed for the histological diagnosis of biliary strictures because transpapillary bile duct biopsy and cytology are more commonly used in practice. However, EUS‐TA may be a viable option when histological diagnosis cannot be obtained via the transpapillary approach; meta‐analyses have demonstrated a high diagnostic yield of EUS‐TA for malignant biliary strictures.^38^, ^39^ Matsumoto *et al*.^40^ reported a case of IgG4‐SC in which histological evidence was successfully obtained using EUS‐TA after multiple transpapillary bile duct biopsies failed to provide a definitive diagnosis. Theoretically, EUS‐TA might be better to obtain tissue sample from bile duct wall stroma than bile duct biopsy because lymphoplasmacytic infiltration and fibrosis of IgG4‐SC are located in bile duct wall stroma with normal epithelium. Given the low sensitivity of bile duct biopsy in diagnosing IgG4‐SC, as previously mentioned, EUS‐TA might be a promising method for obtaining histological evidence of IgG4‐SC. The efficacy of EUS‐TA in the diagnosis of IgG4‐SC has not been elucidated to date. Therefore, further studies are needed to evaluate the yield of EUS‐TA to obtain histological evidence of IgG4‐SC.

Endoscopic ultrasonography‐guided tissue acquisition is essential for the definitive diagnosis of AIP and its differentiation from pancreatic cancer. Although EUS‐guided fine‐needle aspiration historically demonstrated a low diagnostic yield for the histological diagnosis of AIP, recent studies have shown that EUS‐guided fine‐needle biopsy has a higher diagnostic yield for this purpose.^41^, ^42^, ^43^ Consequently, the current role of EUS‐TA is twofold: it serves as an option for obtaining a histological diagnosis of IgG4‐SC and is a critical modality for securing a histological diagnosis of AIP as an OOI of IgG4‐SC.

## ALGORITHM FOR THE ENDOSCOPIC DIAGNOSIS OF IgG4‐SC

**Figure 6:**
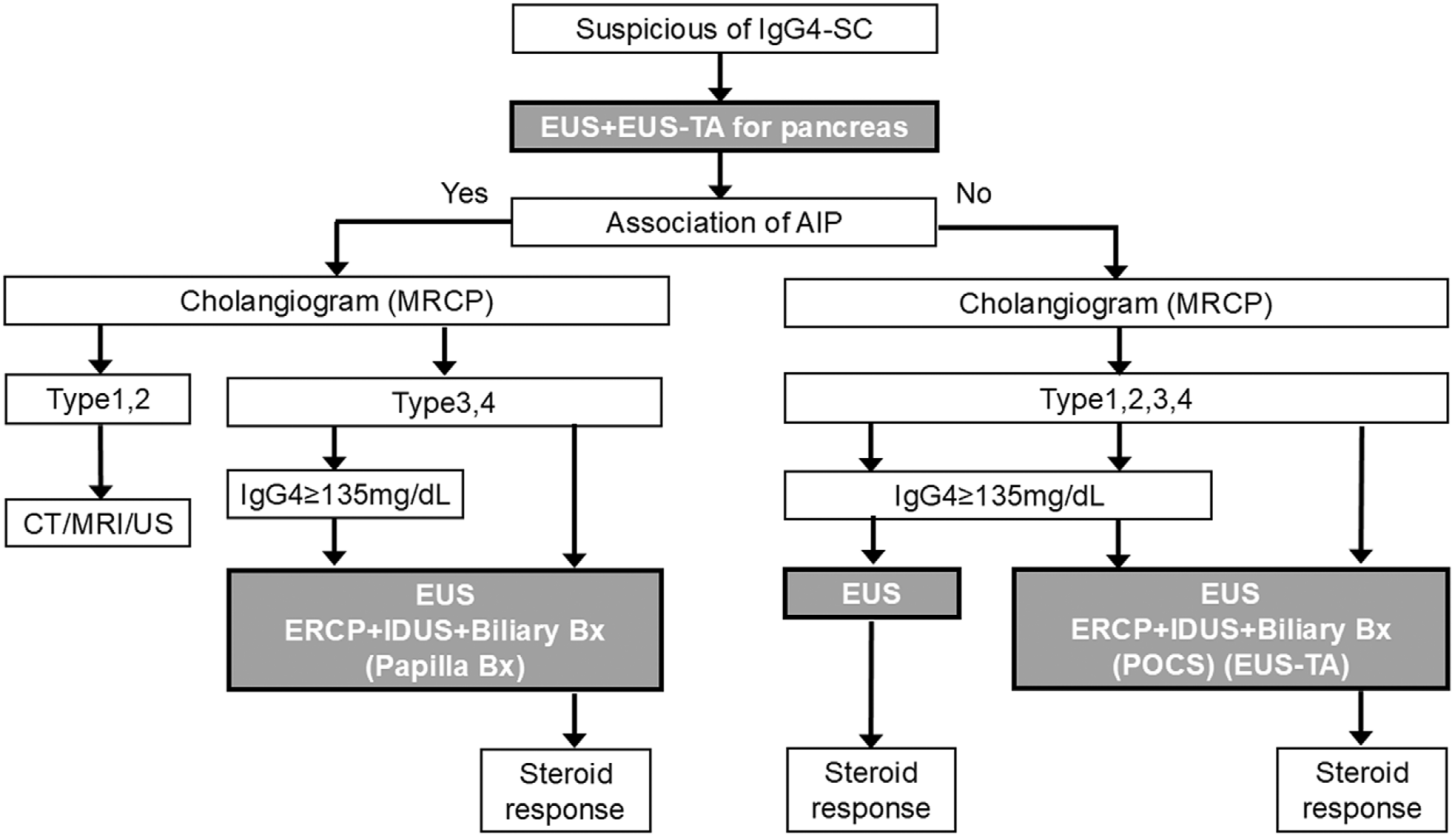
The algorithm for the endoscopic diagnosis of immunoglobulin G4 (IgG4)‐related sclerosing cholangitis (IgG4‐SC). AIP, autoimmune pancreatitis; Bx, biopsy; CT, computed tomography; ERCP, endoscopic retrograde cholangiopancreatography; EUS, endoscopic ultrasonography; EUS‐TA, EUS‐guided tissue acquisition; IDUS, intraductal ultrasonography; MRCP, magnetic resonance cholangiopancreatograpy; MRI, magnetic resonance imaging; POCS, peroral cholangioscopy; US, ultrasonography.

Figure 6 illustrates the algorithm for the utilization of endoscopic modalities in the diagnosis of IgG4‐SC. When IgG4‐SC is suspected, it is crucial to evaluate its association with AIP. EUS and EUS‐TA are valuable modalities for diagnosing AIP. Subsequently, cholangiographic findings should be classified into four types based on the cholangiographic classification in the IgG4‐SC2020 criteria using magnetic resonance cholangiopancreatography.^3^

If the association of AIP is confirmed, the diagnosis of IgG4‐SC becomes relatively straightforward. In type 1 or type 2 IgG4‐SC associated with AIP, endoscopic modality is not mandatory. In type 3 or type 4 IgG4‐SC with AIP, ERCP, EUS or IDUS, and bile duct biopsy are necessary, and duodenal papilla biopsy is optional. The diagnosis of IgG4‐SC in these cases requires a combination of these endoscopic modalities along with evidence of elevated serum IgG4 levels and/or responsiveness to steroid therapy.

Conversely, when AIP cannot be confirmed (isolated IgG4‐SC), the diagnostic process becomes more complex. In cases with elevated IgG4 levels and steroid responsiveness, EUS is essential. In other isolated IgG4‐SC cases, ERCP, EUS or IDUS, and bile duct biopsy are required, and POCS and EUS‐TA are optional. The diagnosis of isolated IgG4‐SC necessitates the integration of these endoscopic modalities with elevated serum IgG4 levels and/or the therapeutic response to steroids. Table 1 summarizes the findings, as well as the advantages and disadvantages, of seven endoscopic modalities used in the diagnosis of IgG4‐SC.

**Table 1:** Findings and pros/cons of endoscopic modalities in the diagnosis of immunoglobulin G4 (IgG4)‐related sclerosing cholangitis

Modality	Findings	Pros	Cons
ERCP	Long/segmental stricture Intrapancreatic stricture	High‐resolution image of biliary stricture	Adverse event of ERCP
IDUS	Circular‐symmetrical wall thickening Preservation of three layers Wall thickening at nonstricture site	High‐resolution image of bile duct wall Successively done after ERCP	Adverse event of ERCP
Bile duct biopsy	Marked lymphoplasmacytic infiltration and fibrosis >10 IgG4(+) plasma cells/HPF	Histological evidence Exclusion of malignancy	Low sensitivity Adverse event of ERCP
POCS	Smooth mucosal surface Dilated vessels without caliber alteration or disruption Absence of easy bleeding	High‐resolution image of bile duct mucosa Exclusion of malignancy Additional bile duct biopsy	High cost Adverse event of ERCP
Duodenal papilla biopsy	Marked lymphoplasmacytic infiltration and fibrosis >10 IgG4(+) plasma cells/HPF	Easy method Higer sensitivity compared to bile duct biopsy	Indirect histological evidence of bile duct
EUS	Bile duct wall thickening	Additional evaluation of pancreas Less invasiveness	Lack of evidence in detailed finding
EUS‐TA	Marked lymphoplasmacytic infiltration and fibrosis >10 IgG4(+) plasma cells/HPF	Histological evidence Additional histological evidence of pancreas	Lack of evidence Risk of dissemination when malignancy

ERCP, endoscopic retrograde cholangiopancreatography; EUS, endoscopic ultrasonography; EUS‐TA, EUS‐guided tissue acquisition; HPF, high‐power field; IDUS, intraductal ultrasonography; POCS, peroral cholangioscopy.

## CONCLUSIONS

We reviewed the current roles of endoscopic modalities in diagnosing IgG4‐SC. ERCP and EUS are the two primary diagnostic approaches for IgG4‐SC. Cholangiographic classification is particularly useful for differentiating IgG4‐SC from its key mimickers, including CCA, PSC, and pancreatic cancer. ERCP‐related modalities, such as IDUS, bile duct biopsy, and duodenal papilla biopsy, play essential roles in the diagnostic process. Similarly, EUS and EUS‐TA are crucial EUS‐related modalities for diagnosing AIP, which is vital for diagnosing IgG4‐SC. Overall, endoscopy serves as a critical tool in the comprehensive diagnosis of IgG4‐SC.

## CONFLICT OF INTEREST

Authors declare no conflict of interest for this article.

## FUNDING INFORMATION

None.
